# A new perspective on balancing life domains: work–nonwork balance crafting

**DOI:** 10.1186/s12889-024-18646-z

**Published:** 2024-04-22

**Authors:** Philipp Kerksieck, Miika Kujanpää, Jessica de Bloom, Rebecca Brauchli, Georg F. Bauer

**Affiliations:** 1 Public and Organizational Health / Center of Salutogenesis, Epidemiology, Biostatistics and Prevention Institute, University of Zurich, Zurich, Switzerland; 2 School of Business, University of South-Eastern Norway, Hønefoss, Norway; 3 Faculty of Economics and Business, University of Groningen, Groningen, The Netherlands; 4 Zurich University of Applied Sciences, Zurich, Switzerland

**Keywords:** Work–life balance, Work–nonwork interface, Life domain boundaries, Employee health, Job crafting, Health-promoting work behaviour

## Abstract

**Background:**

Self-initiated and proactive changes in working conditions through crafting are essential for shaping work and improving work-related well-being. Recently, the research stream of job crafting has been extended to other life domains. The present paper aims to study a novel crafting concept—work–nonwork balance crafting—investigating the role of its antecedents and identifying relevant outcomes. Work–nonwork balance crafting is defined as individuals’ unofficial techniques and activities to shape their work–nonwork balance, here considering their life domain boundary preferences.

**Methods:**

In the study, 1,060 employees in three European countries (Austria, Germany and Switzerland) were surveyed in a longitudinal three-wave study with three-month intervals. We explored the influences of job/home demands and resources as antecedents of work–nonwork balance crafting. Important constructs for employee health and well-being (i.e., work engagement, work-related burnout, mental well-being and detachment from work) were investigated as outcomes.

**Results:**

The findings suggest that resources and demands in the context of work or home are key antecedents of work–nonwork balance crafting. Work–nonwork balance crafting was also predictive for important employee health and well-being outcomes over three months, mainly in a positive and health-promoting way.

**Conclusion:**

This study provides insights into the antecedents of proactive efforts to balance the complex interplay of life domains. By studying work–nonwork balance crafting, we provide a new perspective on crafting beyond job crafting, which may help maintain or improve employees’ mental health and well-being.

**Supplementary Information:**

The online version contains supplementary material available at 10.1186/s12889-024-18646-z.

## Introduction: need, aim and contribution of our study

In recent years, living and working conditions have become ever more flexible and intensified [1, 2]. This development raises research interest in the relevance of work-nonwork balance for employee health outcomes that have been described in earlier research [3]. Built on and extending the well-established concept of job crafting [4, 5] proactive individual-level strategies for balancing life domains and adjusting life domain boundaries are becoming increasingly important for employee mental health, well-being and performance [6–8]. A trend boosted by the COVID-19 pandemic [9]. As a result, crafting efforts capturing both employees’ work and nonwork life domains are being more and more researched [10–13]. Further, extending crafting research towards the interplay of both life domains may help in better understanding how employee resources are restored and demands are balanced, both within and across life domains [6, 10, 14].

Work–nonwork balance crafting (WNBC) [11, 15] refers to an individual’s unofficial techniques and activities to shape work–nonwork balance by considering their life domain boundary preferences. Considering the increasing importance of life domain boundaries for achieving work–nonwork balance [16], WNBC involves adjusting the increasingly permeable and flexible boundary between life domains [7]. Drawing from job demands-resources theory, WNBC may be particularly relevant as an intervening link between demands and resources in the work and nonwork domains, on the one hand, and employee well-being outcomes, on the other hand [17]. We follow the recent and more inclusive shift towards using the term work-nonwork balance instead of work-life balance [11, 18, 19]. It recognizes that individuals engage in various roles and activities beyond their professional, family or care roles. Work-nonwork balance acknowledges that achieving balance involves, for example, managing the demands of work with the wide array of nonwork activities and roles an individual might have, not limited to family or care responsibilities and including roles such as volunteer worker, hobbyist, sporter, or musician. This new perspective allows for a more personalized approach to balance, understanding that what constitutes meaningful or necessary nonwork activities can vary significantly from person to person. It’s a recognition of the complexity of modern lives and the many different aspects that contribute to an individual’s overall well-being.

In the present study, we aim to uncover more about the role of work and nonwork demands and resources for crafting a work–nonwork balance, particularly regarding their promoting or hindering influence on crafting, as outlined, for example, in updated propositions of job demands-resources theory [14]. Furthermore, we are interested in how employees’ WNBC efforts are associated with important employee health and well-being outcomes (work engagement, mental well-being, work-related burnout and detachment from work).

Our study provides several contributions to the literature. First, by integrating the concepts of work–nonwork balance and crafting, we combine these two research streams and add to the understanding of how crafting provides a helpful means for enacting a desired work–nonwork balance. As such, we highlight the scarcely researched interface between the work and nonwork domains as an essential target of employees’ crafting efforts [14], showing that work–nonwork balance can be proactively and intentionally shaped [6].

Second, we address which job/home demands and resources are associated with crafting for work–nonwork balance. This is an important contribution to the crafting literature because job/home demands and resources have rarely been empirically studied as antecedents of crafting, even though job demands-resources theory (proposition 2 [17]) posits that they constitute essential antecedents [14, 17, 20, 21]. In doing so, we add to the literature on the triggers of crafting [6, 20–22] and to recent discussions on crafting as an adaptive strategy in demanding or resource-scarce work–life situations [23]. This perspective provides new knowledge on the role of antecedents of crafting efforts for initiation of resource gain cycles. The Work-Home Resources model [24] proposed the concept of accumulating resources across both work and non-work environments. According to this model, gaining resources in the workplace can initiate a “gain spiral”, leading to enhanced personal resources that can positively impact well-being outside of work (and vice versa). As individuals acquire resources, their resource reservoirs expand, increasing the number of option and likelihood of obtaining further resources in the future.

Third, we focus on the differential relevance of this new crafting concept for a range of key employee health and well-being outcomes (work engagement, mental well-being, work-related burnout and detachment from work). In doing so, we broaden the limited knowledge on crafting one’s work–nonwork balance while providing insights into the nomological net of work–nonwork balance crafting, its antecedents and its outcomes. Finally, we provide an understanding of this new crafting concept relevant to developing interventions that help unify better demanding and boundaryless working conditions and support satisfying work–nonwork balance [24].

The following section provides a more detailed introduction to the work–nonwork balance crafting concept as a suggested extension of the seminal job crafting concept to other life domains.

## Background

### Crafting

Crafting is a proactive, goal-oriented, individual-level behaviour applied to establish a person–environment fit between personal characteristics and those encountered in the work environment (e.g., demand-abilities fit) [25] and satisfy basic psychological needs [6]. Several meta-analyses have found that job crafting has been associated with numerous health and well-being outcomes investigated in our study: job crafting was negatively associated with work-related burnout [26] and positively associated with work engagement [27] and mental well-being [28]. Furthermore, intervention studies on job crafting have reported positive outcomes [29, 30]. However, job crafting might also be associated with detrimental outcomes, such as job strain. Holman et al. [31] provided meta-analytical evidence for such associations when given demands, such as workload or hindering demands, were the target of job crafting efforts.

Moreover, job crafting can either hinder or foster psychological detachment. A recent study identified that job crafting that increased challenging demands decreased detachment [32], providing evidence that job crafting fosters cross- and spillover effects e.g., between life domains. Because of the crucial relevance of crafting for the balance of life domains for employee health and well-being, more research in this area is urgently needed [33]. In the following section, we introduce crafting for balancing life domains that involve their boundaries.

### Work–nonwork balance crafting

Given the often ambiguous definition of work–nonwork balance (WNB) [18] and other instruments aiming to assess crafting for WNB [34, 35], it is essential to set the stage by defining the construct to be crafted. We follow the definition Casper [18] providing a life-domain role-based definition that includes important affective experiences that are omitted elsewhere:

“Employees’ evaluation of the favorability of their combination of work and nonwork roles, arising from the degree to which their affective experiences and their perceived involvement and effectiveness in work and nonwork roles are commensurate with the value they attach to these roles” (p. 197).

Here, we are interested in proactive adjustments in one’s work–nonwork balance, relating to what Sturges [15] defined as ‘the unofficial techniques and activities that individuals use to shape their own work-life balance’ (p. 1540).

As a result, WNBC builds on the pioneering qualitative study by Sturges [15] and can be defined as ‘the unofficial techniques and activities individuals use to shape their own work–nonwork balance under consideration of their boundary preferences and their favoured combination of work and nonwork roles’ [11].

At the core, this construct represents crafting towards a balance of life domain roles [36]. Therefore, crafting as an individual-level construct involves focusing on role demands and opportunities in one of these life domains. Notably, an ideal WNB is what the individual defines as such: ‘Employees’ evaluation of the favourability of their combination of work and nonwork roles, arising from the degree to which their affective experiences and their perceived involvement and effectiveness in work and nonwork roles are commensurate with the value they attach to these roles’ [18]. This ideal balance may be attained through proactivity in the work and nonwork domains. Therefore, WNBC is a two-dimensional construct containing a) WNBC work reflecting crafting one’s WNB with an emphasis on work–life matters and b) WNBC nonwork referring to such crafting emphasising nonwork role characteristics. As shown in the definition, the specific WNBC strategies also consider a person’s preferences for adjusting life domain boundaries by adjusting the interplay between life domains. For example, a person might temporarily set aside a nonwork task to attain a project goal at work or proactively inform one’s social environment that availability and communication outside of work will be reduced for the duration of the work-related project.

WNBC is enacted in three domains identified by Sturges [15]: *Physical crafting* includes techniques like time management, selection and alternation of the work location, such as leaving work early to do necessary personal chores. *Relational crafting* includes managing the quality of relationships during working hours and personal life, for example, explaining to one’s partner that work needs to be extended to meet an urgent deadline that may be achieved by proactively initiating social support [37]. Finally, *cognitive/emotional crafting* refers to framing and redefining WNB in idiosyncratic terms, intentionally prioritising work or nonwork at the expense of the respective other life domain and compromising an ideal WNB in return for long- and short-term benefits [15]. In developing the WNBC scale, Sturges’ [15] dimension of cognitive crafting was renamed cognitive/emotional crafting and integrated the emotional aspects because these are particularly relevant for work–nonwork conflicts and enrichment [38, 39], for example, in preventing negative emotional states from intruding in and affecting another life domain. This is an essential aspect of how the WNBC scale differs from the work–life balance crafting behaviours survey [34], which does not consider this emotional dimension. This is a relevant aspect of WNB that is reflected in Casper’s [18] seminal definition of WNB, where affective experiences in work and nonwork roles are emphasised. Moreover, an important extension to previous approaches to measure WNBC [34] is the two-dimensional structure of the WNBC scale [11], which allows the respective crafting efforts for each life domain to be assessed.

The results from longitudinal validation studies of the two-dimensional WNBC scale have indicated that WNBC was positively associated with job and family role performance, subjective vitality, job/life satisfaction and work–nonwork balance [11]. However, this previously study did not investigate demands and resources, leaving it unclear how the potential key antecedents of demands and resources at work or home either promote or hinder crafting for WNB.

### The relevance of job/home demands and resources for work–nonwork balance crafting

Resources and demands holistically refer to anything that helps or hinders an individual in attaining a goal [40]. Drawing from conservation of resources theory [41], it has been shown that the available resources motivate job crafting and increase work engagement [42]. Resources, as conceptualised in conservation of resources theory, are not limited to job-related factors. The work–home resource model describes the interplay between the two domains of life as either conflicting with each other or enriching each other, depending on the resources available. Therefore, we argue that resources in both life domains increase the likelihood of engaging in crafting for the balance of life domains. This aligns with the perspective on resources taken in the new propositions of job demands-resources theory, which integrates various types of demands and resources beyond the job [14].

Concerning the role of demands across both life domains, we again build on the propositions of job demands-resources theory. Therefore, demands are proposed to be positively associated with strain (e.g., exhaustion, health complaints). Furthermore, demands buffer the positive association of resources with motivation (e.g., work engagement, flourishing). With this, they inhibit the motivation to craft. We expect these mechanisms for demands and resources to occur in work [21] and in the nonwork domain [10].

Apart from theoretical claims, empirical evidence is scarce. Despite cross-sectional and short-term associations between job–home demands and resources and crafting [e.g., 10, 13, 43, 44], only a few studies have investigated the long-term (e.g., monthly or yearly) effects of job–home demands and resources as antecedents of crafting. For example, Mäkikangas et al. [45] reported that self-efficacy, team climate and perceptions of connecting leadership are positively associated with team job crafting. Further, Petrou et al. [46] found that work pressure and autonomy were positively associated longitudinally with job crafting. In the off-job domain, Petrou and Bakker [13] examined the link between home demands and resources and leisure crafting; they found that the interaction between home autonomy and quantitative home demands was unrelated to leisure crafting.

The first research question addresses the role of demands and resources as hindering or facilitating factors of WNBC. This will also help in understanding whether crafting is a strategy for adapting to demanding situations and/or a strategy that allows cultivating resources [23]. Based on the above-outlined reasoning, we formulate the following hypotheses.

#### Hypothesis

*a: Job and home resources elicit WNBC.*

#### Hypothesis

*b: Job and home demands inhibit WNBC.*

### The relevance of crafting for health and well-being outcomes

Our second main research interest addresses the differential relevance of WNBC as a new crafting concept for important employee health and well-being outcomes. In other words, does engaging in proactively shaping WNB promote employee health and well-being? We introduce these studied outcomes by referring to meta-analytical evidence reporting on these concepts.

#### Work engagement

Work engagement is defined as ‘a fulfilling work-related state of mind that is characterised by vigour, dedication and absorption’ [47]. It is consistently related to occupational health [48] and organisational outcomes [49]. Job crafting has been identified as a positive predictor of work engagement [27, 50]. WNBC work emphasises attaining work-related goals, so we expect it to be positively related to work engagement. WNBC nonwork focuses on private life roles and goals, which may temporarily reduce one’s degree of involvement at work. As such, we expect WNBC nonwork to be negatively, rather than positively, associated with work engagement.

*We derive the following hypotheses*:

##### Hypothesis

*a: WNBC work is positively related to work engagement.*

##### Hypothesis

*b: WNBC nonwork is negatively related to work engagement.*

#### Mental well-being

Mental well-being can be understood as ‘Hedonic and eudaimonic aspects of mental health including positive affect (…), satisfying interpersonal relationships and positive functioning (…)’ [51]. Job crafting has been shown to affect well-being directly or through mediation processes, for example, by satisfying psychological needs [23, 52]. WNBC is assumed to lead to an increase in fit with life perspectives and, thus, positive affect, as well as to positive functioning in both the work and nonwork domains.

*We derive the following hypotheses*:

##### Hypothesis

*a: WNBC work is positively related to mental well-being.*

##### Hypothesis

*b: WNBC nonwork is positively related to mental well-being.*

#### Work-related burnout

Work-related burnout is defined as ‘the degree of physical and psychological fatigue and exhaustion that is perceived by the person as related to his/her work’ [53]. Lichtenthaler and Fischbach [26] found mixed associations between different types of job crafting and burnout. In a study by Hakanen et al. [43], job crafting buffered the adverse effects of job demands on burnout. Balancing work and nonwork life domains in accordance with one’s own needs and standards through WNBC may balance the detrimental effects of work strain, decreasing burnout because the employee creates a better harmony and fit between their work and nonwork roles [19].

*We derive the following hypotheses*:

##### Hypothesis

*a: WNBC work is negatively related to work-related burnout.*

##### Hypothesis

*b: WNBC nonwork is negatively related to work-related burnout.*

#### Detachment from work

Psychological detachment from work refers to mental disengagement from work during off-job hours [54]. Detachment is positively associated with a wide variety of indicators of health and well-being, such as sleep, positive affect and state of recovery [55]. Despite the crucial importance of detaching from work-related thoughts and activities for sustainable occupational health [56], earlier research has overlooked the role of WNBC in this regard. The subdimension WNBC nonwork, which aims to better satisfy needs in the nonwork domain, is assumed to positively contribute to detachment [6]. In contrast, WNBC work focusing on the work domain could make disengaging from unfinished work challenging and lead to detachment difficulties [57].

*We derive the following hypotheses*:

##### Hypothesis

*a: WNBC work is negatively related to detachment from work.*

##### Hypothesis

*b: WNBC nonwork is positively related to detachment from work.*

## Methods

### Study design, sample and procedure

We used a panel survey design with a variable-centred approach among a sample of employees from Austria, Germany and Switzerland. The survey was conducted at three measurement points at three-month intervals from December 2018 to June 2019. The study design meets the recommendation of Rauvola et al. [58] to integrate at least three measurement points for considering lagged relationships and change [59]. It also considers Sanchez and Viswesvaran’s [60] recommendation to measure predicting variables before outcomes and Dormann and Griffin’s [61] propositions to use relatively short time lags for panel studies. We invited 3,232 employees to participate via an online panel service provider. The participants provided their written informed consent to participate in this study. The inclusion criteria were gainfully employed individuals working more than 20 h per week and aged 18–65 years. The resulting sample at T1 comprised 2,104 individuals; at T2, three months later, 1,503 of these individuals took part (71.4%), and 1,160 participants completed all three waves (55.2% of the T1 sample). We also tested for nonrandom sampling using multiple logistic regression [62]. See Appendix A for further details.

This final sample was distributed as follows: Germany 69.5%, Austria 17.7% and Switzerland 12.8%. The participants had a mean age of 45.41 years (*SD* = 10.56), and 52% identified as male. The average weekly working time was 40–49 h, and almost all participants were employed by a single employer (94.4%) with a mean organisational tenure of 11.94 years (*SD* = 10.15). They worked in a broad range of economic sectors and occupations, including health care and social work (13.5%), public administration (11.9%) and education (7.5%). Further, 43.8% had completed an apprenticeship, and 29.9% had earned a degree from a higher educational institution.

### Measures

#### Job demands and resources

We applied the United Kingdom Health and Safety Executive Management indicator tool [63]. Eight items were used to assess job demands and sample items: ‘I am pressured to work long hours’ or ‘I have to work very intensively’. In addition, we used the Salutogenic Subjective Work Analysis questionnaire [64]. Three items covering qualitative work overload were surveyed: ‘It happens that work is too difficult for oneself’. Of the indicator tool, we assessed 20 items covering the following resource dimensions: work colleague support ‘I get the help and support I need from colleagues’, managerial support ‘I am given supportive feedback on the work I do’, job control ‘I have a choice in deciding how I do my work’ and role ‘I am clear of what is expected of me at work’. Three items of the Salutogenic Subjective Work Analysis questionnaire referring to resources were used, for example, ‘This work creates good opportunities to progress in the job’. All items were answered on a 5-point scale ranging from 1 = ‘I do not agree at all’ to 5 = ‘I fully agree’.

#### Home demands and resources

The full eight-item Home Demands Scale was used to capture home demands, which relates conceptually to the Job Demands Scale [65]. The Home Demands Scale captures quantitative (‘Do you find that you are busy at home?’), emotional (‘How often do emotional issues arise at home?’) and mental home demands (‘Do you have to do many things simultaneously at home?’). Home resources were measured with 11 items of the Home Resources Instrument [66], referring to home autonomy (‘I have control over how I use my free time’), home social support (‘My partner/family help(s) me with a certain task if necessary’) and home developmental possibilities (‘I can develop my talents during my free time’). Answers for both scales ranged from 1 = ‘never’ to 5 = ‘always’.

#### Work–nonwork balance crafting

The WNBC scale [11] covers the dimensions of WNBC work and WNBC nonwork. Each dimension contains eight items answered on a 5-point scale ranging from 1 = ‘I do not agree at all’ to 5 = ‘I fully agree’. Sample items for WNBC work read, ‘When I must get some work chores done, I come home later or go to work earlier, if necessary’ (physical crafting), ‘When I’m in a bad mood because of personal matters, I try not to let this affect my work environment’ (cognitive/emotional crafting) and ‘I tell people in my private environment when I’m unable to communicate with them during working time or to take care of private matters’ (relational crafting). For WNBC nonwork, the scale uses similar item wordings to reflect analogous crafting techniques in the nonwork life domain, for example, ‘When I must get some personal chores done, I come to work later or go home earlier, if necessary’.

#### Work engagement

We used the Utrecht Work Engagement Scale (UWES-9; [47]) to measure work engagement. This scale covers the three subdimensions of vigour, dedication and absorption. The scale contains nine items answered on a 7-point scale from 0 = ‘never’ to 6 = ‘always’.

#### Mental well-being

The Warwick–Edinburgh Mental Well-Being Scale covers both hedonic (happiness and life satisfaction) and eudemonic (functioning, relationships and agency) well-being within the past two weeks [51]. We used the seven-item short version of the Warwick–Edinburgh Mental Well-Being Scale [67] with a scale ranging from 1 = ‘none of the time’ to 5 = ‘all of the time’.

#### Work-related burnout

The seven-item Copenhagen Burnout Inventory subdimension of work-related burnout was applied [53] to assess the physical and psychological exhaustion related to work. Items were answered on a 5-point scale from 1 = ‘never/almost never’ to 5 = ‘very often’.

#### Detachment from work

To measure detachment from work, the item ‘I don’t think about work at all’ from the Recovery Experience Scale [68] was used. This item closely matches the core idea behind detachment, namely ‘leaving the workplace behind oneself in psychological terms’ [68]. The item was answered on a 5-point scale from 1 = ‘I do not agree at all’ to 5 = ‘I fully agree’.

### Analytical approach

**Fig. 1:**
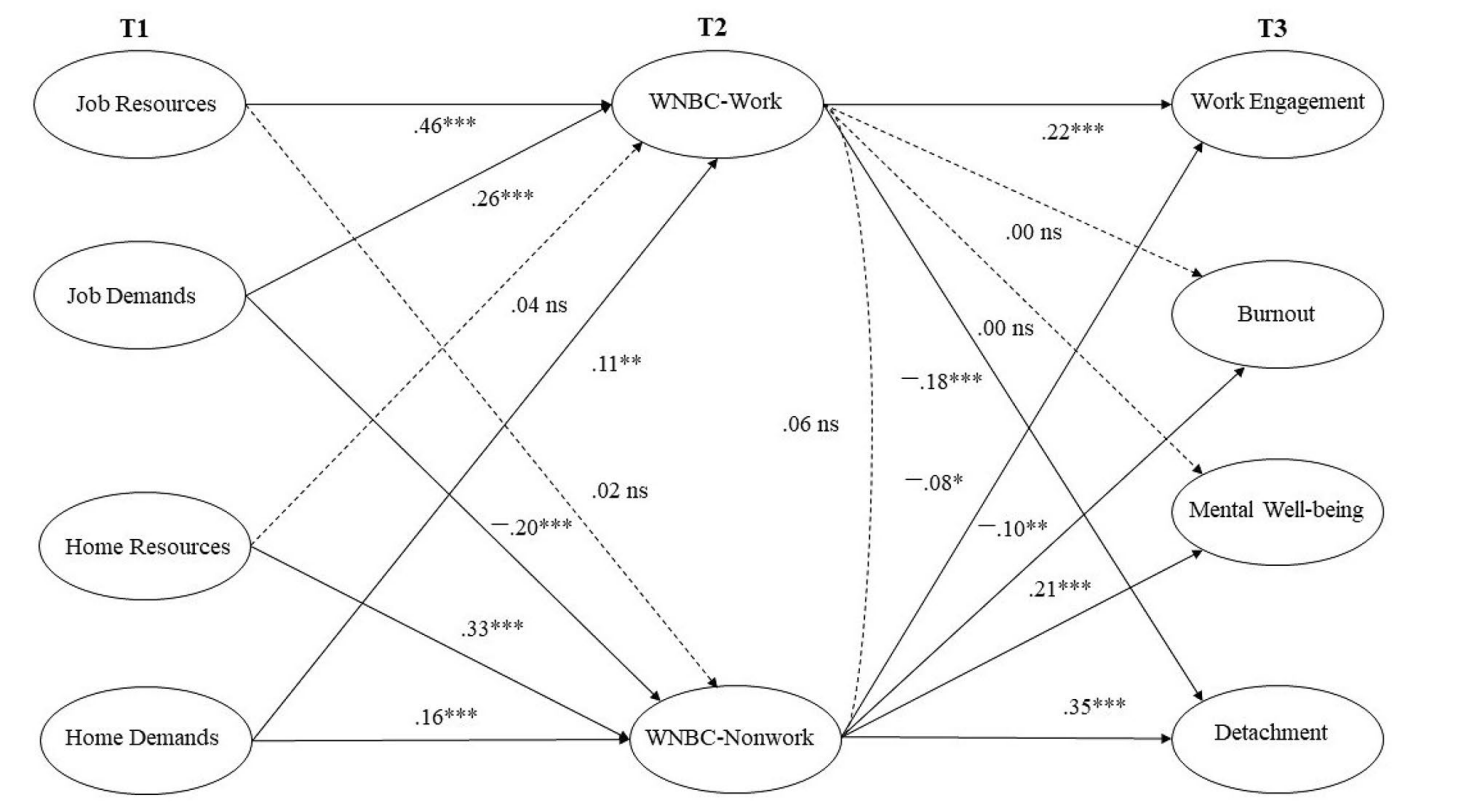
Standardised estimates of structural equation modelling testing the time-lagged model of job/home resources and demands at T1, WNBC-work/nonwork at T2 and work engagement, work-related burnout, mental well-being and detachment from work at T3. Significant and non-significant paths (broken lines) are depicted in the diagram. * *p* < .05; **p < .01; ***p < .00; *ns* = not significant

For analysing the data, we followed the suggestions and a comparable approach of the time-lagged structural equation model and for contextualizing WNBC in a larger nomological net [69]. A time-lagged structural equation model assessed job–home demands and resources at T1, predicting WNBC and at T2 and predicting the outcomes at T3 (Fig. 1). In addition, resources and demands were regressed on the four studied outcomes (Table 2). We did not assess autoregressive effects because we focused on exploring the associations between concepts, not the effects of the constructs on themselves over time. This allows for integrating a broad spectrum of antecedents and outcomes without overloading the model. Each item was individually incorporated into the structural equation model and linked to its corresponding latent construct rather than combining items into mean or summed scores or item parcels. Latter practice may lead to a loss of information on item level and relative item importance [70]. It may further underestimate associations between latent variables [71]. For an application of a related structural equation modelling approach in crafting research, see Petrou et al. [72]. Error variances were correlated through an iterative, item-by-item process. These correlations were specifically constrained to reflect the underlying, common latent construct they represent (for example, mental well-being), as supported by both theoretical frameworks and the research design [73].

The analysis was performed using IBM SPSS 26 and IBM AMOS 26 [74]. Indices for model fit assessment were comparative fit index (CFI), incremental fit index (IFI) and root mean square error of approximation (RMSEA) with 90% confidence interval (CI) [75] and PClose. The cutoff values for CFI and IFI were 0.90 [76] and RMSEA 0.06 [77]. We also calculated the Chi-square/df ratio (*χ²*/*df*), for which good fits would be *χ²*/*df* < 3 [78].

#### Preparatory analysis

The present study was conducted with time-lagged measurements to avoid common method variance [79]. In testing for this precondition, a post hoc Harman’s single-factor test was performed with the study variables at T1 to ascertain if the eigenvalues produced by the first factor exceeded 50% of the total variance; the test revealed that the unrotated factor accounted for 32.1% of the variance. Thus, common method variance did not affect our data [80].

## Results

### Correlations between the concepts studied

Table 1 reports the means, standard deviations, correlations and scale reliabilities. The bivariate correlations indicated that resources in both life domains were significantly positively correlated with WNBC, ranging from *r* = .12 to *r* = .32. Demands in both life domains were all significantly negatively or positively correlated with the WNBC dimensions, ranging from *r* = − .12 to *r* = .16.

**Table 1:** Means, Standard Deviations, Pearson’s Correlations and Cronbach’s Alphas at T1 (between brackets on the diagonal) among the study variables

		M	SD		1		2	3		4	5	6		7		8	9	
1.	Job resources (T1)	3.58	0.59		(0.90)													
2.	Job demands (T1)	2.52	0.71		−0.27**		(0.86)											
3.	Home resources (T1)	3.38	0.71		0.36**		−0.20**	(0.86)										
4.	Home demands (T1)	2.88	0.72		−0.05		0.28**	−0.14**		(0.88)								
5.	WNBC-Work (T2)	3.59	0.58		0.32**		0.12**	0.12**		0.16**	(0.66)							
6.	WNBC-Nonwork (T2)	3.56	0.57		0.20**		−0.12**	0.26**		0.08**	0.28**	(0.67)						
7.	Work engagement (T3)	2.85	1.33		0.56**		−0.20**	0.28**		−0.01	0.35**	0.05		(0.97)				
8.	Mental well-being (T3)	3.70	0.66		0.39**		−0.31**	0.44**		−0.19**	0.11**	0.23**		0.49**		(0.89)		
9.	Work-related burnout (T3)	2.52	0.85		−0.48**		0.52**	−0.29**		0.25**	−0.06*	−0.15**		−0.59**		−0.62**	(0.90)	
10.	Detachment from work (T3)	3.45	1.10		0.15**		−0.29**	0.14**		−0.14**	−0.10**	0.29**		0.08**		0.30**	−0.36**	

*Note. N* = 1,160

T1 = Study wave 1; T2 = Study wave 2; T3 = Study wave 3.

* *p* < .05; ** *p* < .01; *** *p* < .001

### Time-lagged structural equation models

As outlined above, we conducted a time-lagged structural equation model to investigate the associations of all the introduced concepts. In the first step, data were tested for multivariate normal distribution, which was not fulfilled. Therefore, we reported fit indices based on robust maximum likelihood estimation. Calculated regression weights were included in Fig. 1. For a concise presentation of the results, not all structural equation model parameters are depicted in Fig. 1 but are included in Table 2.

**Table 2:** Results of structural equation modelling referring to the model in Fig. 1

			β	SE	b	p
**T1** → **T2**						
JR T1	→	WNBC-work T2	0.46	0.02	0.13	***
JR T1	→	WNBC-nonwork T2	0.02	0.02	0.00	0.71
JD T1	→	WNBC-work T2	0.26	0.01	0.07	***
JD T1	→	WNBC-nonwork T2	−0.20	0.02	−0.10	***
HR T1	→	WNBC-work T2	0.04	0.01	0.01	0.34
HR T1	→	WNBC-nonwork T2	0.33	0.03	0.17	***
HD T1	→	WNBC-work T2	0.11	0.01	0.03	**
HD T1	→	WNBC-nonwork T2	0.16	0.02	0.09	***
**T1** → **T3**						
JD T1	→	WE T3	−0.14	0.04	−0.21	***
JD T1	→	MWB T3	−0.14	0.02	−0.10	***
JD T1	→	DFW T3	−0.16	0.05	−0.20	***
JD T1	→	BOUT T3	0.35	0.03	0.33	***
JR T1	→	WE T3	0.54	0.06	0.81	***
JR T1	→	MWB T3	0.26	0.03	0.21	***
JR T1	→	DFW T3	0.10	0.06	0.14	***
JR T1	→	BOUT T3	−0.38	0.04	−0.39	***
HD T1	→	WE T3	0.03	0.04	0.05	0.21
HD T1	→	MWB T3	−0.12	0.03	−0.10	***
HD T1	→	DFW T3	−0.09	0.04	−0.12	***
HD T1	→	BOUT T3	0.14	0.03	0.14	***
HR T1	→	WE T3	0.06	0.05	0.09	0.05
HR T1	→	MWB T3	0.30	0.03	0.24	***
HR T1	→	DFW T3	−0.04	0.05	−0.05	***
HR T1	→	BOUT T3	−0.06	0.32	−0.06	0.09
**T2** → **T3**						
WNBC-work T2	→	WE T3	0.22	0.22	1.15	***
WNBC-work T2	→	BOUT T3	0.00	0.10	0.00	0.98
WNBC-work T2	→	MWB T3	0.00	0.09	0.00	0.96
WNBC-work T2	→	DFW T3	−0.18	0.13	−0.82	***
WNBC-nonwork T2	→	WE T3	−0.08	0.10	−0.22	*
WNBC-nonwork T2	→	BOUT T3	−0.10	0.06	−0.19	**
WNBC-nonwork T2	→	MWB T3	0.21	0.06	0.33	***
WNBC-nonwork T2	→	DFW T3	0.35	0.13	0.91	***

*Note. N* = 1,160,

* *p* < .05; ** *p* < .01; *** *p* < .001

JR = job resources, JD = job demands, HR = home resources, HD = home demands, WNBC-work = work-nonwork balance crafting-work, WNBC-nonwork = work-nonwork balance crafting-nonwork. WE = work engagement, MWB = mental well-being, DET = detachment from work, BOUT = burnout; T1 = Study wave 1; T2 = Study wave 2; T3 = Study wave 3

The fit between the data and these models displays the following results for WNBC: *χ*² (3918) = 8757.254, *p* < .001; *χ²*/*df* = 2.24; CFI = 0.918; RMSEA = 0.033, 90% CI = [0.032, 0.034]; PClose = 1.000; IFI = 0.918.

Referring to Hypothesis 1a, job resources at T1 were significantly positively related to WNBC work at T2 (*B* = 0.46, *p* < .001) but unrelated to WNBC nonwork at T2 (*B* = 0.02, *p* = .71). Home resources at T1 were significantly positively related to WNBC nonwork at T2 (*B* = 0.33, *p* < .001) but not to WNBC work at T2 (*B* = 0.04, *p* = .34), partially confirming Hypothesis 1a.

Regarding Hypothesis 1b, the job demands at T1 were positively related to WNBC work at T2 (*B* = 0.26, *p* < .001) and negatively associated with WNBC nonwork at T2 (*B* = − 0.20, *p* < .001). The home demands at T1 were positively related to WNBC work at T2 (*B* = 0.11, *p* < .01) and to WNBC nonwork at T2 (*B* = 0.16, *p* < .001). Again, Hypothesis 1b was partially confirmed.

Taken together, the results relating to Hypothesis 1a and 1b offer a differentiated picture: on the one hand, job resources, home resources and home demands elicited both WNBC dimensions, whereas job demands were positively related to WNBC work but negatively related to WNBC nonwork, suggesting systematic variations of crafting efforts because of posed demands and resources available.

Concerning the potential outcomes of WNBC (Table 2), we obtained the following results: WNBC work at T2 was positively related to work engagement at T3 (*B* = 0.22, *p* < .001) and negatively related to detachment from work at T3 (*B* = − 0.18, *p* < .001), providing support for Hypotheses 2a and 5a. WNBC work at T2 was unexpectedly not significantly related to work-related burnout at T3 (*B* = 0.00, *p* = .98) or mental well-being at T3 (*B* = 0.00, *p* = .96). Thus, Hypotheses 4a and 3a were rejected. WNBC nonwork at T2 was positively related to mental well-being at T3 (*B* = 0.21, *p* < .001) and detachment from work at T3 (*B* = 0.35, *p* < .001), providing support for Hypotheses 3b and 5b. WNBC nonwork at T2 was negatively related to work engagement at T3 (*B* = − 0.08, *p* < .05) and work-related burnout at T3 (*B* = − 0.10, *p* < .01), providing support for Hypotheses 2b and 4b.

To establish a complete framework of all studied constructs, the direct association between job/home demands (T1) and resources with health or well-being constructs (work engagement, mental well-being, work-related burnout, and detachment from work) (T3) will be studied. The nature of these associations provides additional contextual information (Table 2):

Job demands at T1 were significantly associated at T3 with work engagement (*B* = − 0.14, *p* < .001), with mental well-being (*B* = − 0.14, *p* < .001), detachment from work (*B* = − 0.16, *p* < .001) and work-related burnout (*B* = 0.35, *p* < .001).

Job resources at T1 were significantly associated at T3 with work engagement (*B* = 0.54, *p* < .001), with mental well-being (*B* = 0.26, *p* < .001), detachment from work (*B* = 0.10, *p* < .001) and work-related burnout (*B* = − 0.38, *p* < .001).

Home demands at T1 were associated at T3 with work engagement (*B* = 0.03, *p* = .21), with mental well-being (*B* = − 0.12, *p* < .001), detachment from work (*B* = − 0.09, *p* < .001) and work-related burnout (*B* = 0.14, *p* < .001).

Home resources at T1 were associated at T3 with work engagement (*B* = 0.06, *p* = .05), with mental well-being (*B* = 0.30, *p* < .001), detachment from work (*B* = − 0.04, *p* < .001) and work-related burnout (*B* = − 0.06, *p* = .09).

We further provide regression parameter controlling for baseline WNBC dimensions at T1 with WNBC dimensions at T2. WNBC-work at T1 was positively related (*B* = 0.65, SE = 0.02; *p* < .001) and WNBC-nonwork at T1 was unrelated (*B* = − 0.03, SE = 0.03; *p* = .30) to WNBC-work at T2 (*R*^2^ = 0.44, *F*(2,1157) = 446.83, *p* < .001). WNBC-work at T1 was unrelated (*B* = − 0.01, SE = 0.02; *p* = .64) and WNBC-nonwork at T1 was positively related (*B* = 0.60, SE = 0.03; *p* < .001) to WNBC-nonwork at T2 (*R*^2^ = 0.33, *F*(2,1157) = 287.22, *p* < .001).

## Discussion

Much is already known about crafting, but most research on crafting has been focusing on job crafting [23, 27]. The present study aimed to investigate the potential antecedents and outcomes of new ways of crafting, namely WNBC [11]. We explored WNBC with a comprehensive time-lagged three-wave study design, surveying 1,060 employees in three European countries. First, we discuss the antecedents and outcomes of WNBC. This is followed by an outline of the practical implications for promoting crafting in employees and organisations. Finally, we present the study’s limitations and propose avenues for future research and organisational practice.

### Job/ home demands and resources as antecedents of WNBC

For WNBC work and WNBC nonwork, demands and resources function predominantly as domain-specific facilitators. Job demands and resources facilitate WNBC work. In contrast, home demands and resources elicit WNBC nonwork.

In addition, demands also activate WNBC across domains. Home demands were linked to more WNBC work crafting, indicating that work is protected against the demands of the nonwork domain through crafting. In contrast, job demands were related to less WNBC nonwork crafting, a cross-domain impediment to crafting in private life. Both findings imply that work-related concerns take precedence over nonwork life, likely because of values that reflect work prioritisation over other life domains. Leslie et al. [81] outlined the importance of values forming ideologies on how people think about how work and life outside work should be related. For example, work-priority perspectives are driven by contextual factors, such as labour market situations. The finding that job demands impede WNBC nonwork crafting is critical because it could result in the depletion of resources or even burnout over time [65, 82] because crafting for the nonwork domain is essential for resource enhancement [12]. Indeed, we found that job demands at T1 were associated with burnout at T3 (Table 2). Furthermore, focusing primarily on work can be assumed to result in neglecting psychological needs satisfaction in the nonwork life domain, for example, the needs for detachment and relaxation [6]. Thus, this type of neglect can likely spill over detrimentally into the work domain, leading to exhaustion.

### Health and well-being as outcomes of work–nonwork balance crafting

For this exploration, we selected a broad set of relevant employee well-being and health indicators to provide a holistic picture of both dimensions of WNBC, comparable to explorative studies on job crafting [83]. These outcomes are discussed in the following sections.

WNBC work was strongly positively associated with work engagement three months later, as we anticipated. Devoting more attention and resources to the work role facilitates engagement in it. WNBC work contributes to work engagement, thus potentially *adding to job crafting*, which, similar to WNBC work, is linked to job demands-resources and associated with work engagement [84].

In contrast, WNBC nonwork (emphasising optimal role performance in the nonwork life domain over the work domain) was negatively associated with work engagement because it partially reduced the resources devoted to engaging in the work domain. Despite its adverse effects on work engagement, WNBC nonwork could be more beneficial for motivation and engagement in other life domains, such as intrinsic leisure motivation [85].

Regarding detachment from work, WNBC work was decidedly negatively associated with this outcome. Because investing crafting efforts in work-related aspects of life roles led to work engagement, this high involvement in the work–life domain seems to also impede detachment from work. However, in our study, this was not associated with higher levels of burnout.

In contrast, WNBC nonwork seems to be a strategy helping employees experience detachment from work. Notably, to the best of our knowledge, this is one of the first studies to examine the recovery experience of detachment from work as a potential outcome of crafting. Detachment is a key enhancer of employee well-being [55, 56]. Successful WNBC nonwork efforts may relieve stressful work-related thoughts by helping employees focus more intensely on personally valuable aspects of their nonwork lives, such as creating flow experiences and a sense of purpose through more satisfying nonwork activities. Overall, this may help detach from work during nonwork time. Besides being a positive outcome in and of itself, detachment also has a catalytic effect on further parameters of employee well-being, such as a positive mood and low fatigue [54]. This result connects the perspectives of recovery as a process (proactively engaging in WNBC nonwork) with recovery as a state that involves feeling detached from work [86].

WNBC work helped employees focus on work-related tasks (improving work engagement) without impairing their mental health because their work-related burnout levels remained unchanged. In the case of job crafting, a protective effect against work-related burnout has even been found [43]. We did indeed find such a protective effect for WNBC nonwork because it was negatively associated with work-related burnout. As shown above, balancing work and nonwork and proactively focusing on the nonwork domain leads to detachment from work, which may protect employees against negative cycles towards work-related burnout [54, 87].

WNBC nonwork was related to improved mental well-being, probably by helping balance work and nonwork in a way that is consistent with work–life values [81], one’s corresponding role definitions [18] and life domain boundaries. Furthermore, considering nonwork life experiences and related roles as important has been associated with mental well-being [88], counteracting potentially excessive work prioritisation [81]. This may also explain why we did not find the anticipated positive relationship between WNBC work and mental well-being because this form of crafting implies a one-sided focus on the work–life domain.

Overall, these findings support earlier work highlighting the role of proactivity in buffering exhaustion and burnout [89]. Proactively shaping the work–nonwork boundary may specifically promote cross-domain spill over and stabilise potential resource expenditure, as suggested by the integrative needs model of crafting [6]. This model stresses the importance of nonwork life experiences for the quality of life in the work domain. In this sense, the results of our study are in line with evidence for the concept of leisure crafting [8].

### Relevance of job/home demands and resources for health and well-being

Our findings indicate that demands across both life domains were generally perceived as detrimental by employees, significantly decreasing work engagement, mental well-being, and reducing detachment from work, while increasing work-related burnout. Conversely, resources in both job and home contexts were predominantly associated with positive outcomes for health and well-being. These results serve as evidence supporting the longitudinal validity of the constructs as they were implemented and statistically modelled.

Interestingly, resources at home did not significantly or substantially translate into work-related experiences such as work engagement or work-related burnout, and they were associated with a minor, albeit significant, negative impact on detachment from work. This observation is interesting for understanding a key focus of our study, as WNBC-nonwork was significantly and positively related to detachment from work and a reduction in work-related burnout. This suggests that crafting plays a catalytic role in the activation of resources and may be a core contributor to the initiation of gain spirals across life domains [6, 90].

Furthermore, these findings prompt a re-evaluation of how life and work conditions should be characterized to facilitate a better transfer of nonwork resources to work-related outcomes. The settings of life-domain boundaries may be an essential factor [91], enhancing the effectiveness of crafting efforts.

In summary, the present study showed that demands and resources are relevant for WNBC primarily at a life domain–specific level. Regarding outcomes, WNBC nonwork was consistently and beneficially associated with the full spectrum of outcomes studied: work-related engagement and (lower) burnout levels, detachment from work and general well-being. The same was found for WNBC nonwork, except for the observed negative relationship with work engagement. Because WNBC nonwork is particularly strongly associated with detachment from work, this type of crafting effort may gain importance attention where life domain boundaries are eroding. In this way, WNBC nonwork is assumed to counteract potentially strenuous work prioritisation. Importantly, the findings provide a strong case for further research and application of proactive efforts to balance life domains.

### Practical implications for crafting interventions

The present research has important individual and organisational implications. WNBC can be applied as an individual or organisational practice to reach an individually chosen balance of work and nonwork [84]. Although any type of crafting can be performed by individuals with limited organisational support, all of these crafting strategies will likely be more effectively and frequently applied when organisations encourage and support them [92]. In this process, organisations should consider the individual preferences of their employees for shaping work and nonwork and open the organisational culture and policy to this diversity [7, 93].

Importantly, having a good work-nonwork balance is associated with employee health. A comparative study on work-life conflict among employees in European welfare states revealed a work-life ‘imbalance’ among employees [94]. It is, therefore, a matter of public health interest to provide measures for proactively reducing life domain conflicts and increasing work-nonwork balance since it is known that they relate to many health problems, including poor physical or mental health and lower life satisfaction [95].

Because crafting has positive outcomes but potentially transfers responsibilities for well-being to the employee, which can be resource-demanding [23, 26], crafting interventions should be complemented with organisational-level improvements in psychosocial working conditions. Nevertheless, developing interventions involving WNBC seems a promising avenue for improving employees’ work–nonwork balance in organisations. Employees will find it helpful to attain such programmes for (a) identifying individual needs regarding crafting and work–life balance and (b) learning to apply crafting efficiently. As suggested for job crafting interventions [92, 96], a stepwise approach can be useful for employees to learn how to apply and implement crafting. These formats may be translated into interventions for life domain balance.

Particularly in the context of the severe impact of the COVID-19 pandemic on working conditions [97] and rapid increase in working from home, in its aftermath, the balance of work and nonwork should be deliberately crafted [98]. It may be important to keep a WNB that ensures recovery from work or staying focused and energised in demanding work to nonwork situations [99].

### Limitations and avenues for future research

Despite the contributions made, the present study also has limitations that need to be addressed in the future.

First, we did not statistically model every variable studied at each survey time point to avoid overloading the model, a procedure conducted and approved earlier [69]. Therefore, we cannot assess the causal directions of the relationships. Future longitudinal research should ascertain the causality between concepts, potentially reversed causality and autoregressive stability over time. It may be possible that autoregressions of potentially stable constructs influence the model parameter. How and under what preconditions can integrated concepts complement and amplify each other, potentially resulting in gain cycles, as shown for job crafting [91], would also merit scrutiny.

Second, internal consistency has been found to be low within the two WNBC subdimensions. However, we encompassed a broad range of WNB crafting efforts (physical, cognitive/emotional, relational crafting) based on the qualitative research by Sturges [15] and validated by Kerksieck et al. [11]. This might have led to lower reliability in the subscales. Nevertheless, the scale meets the purpose of representing conceptual breadth within the WNBC construct rather than maximising its internal consistency.

Third, we focused on job and home demands and resources as relevant and meaningful indicators of everyday life [63–65]. However, further antecedents or contextual factors influence crafting. Cultural practice [28], task and social context [83] and crafting orientation towards avoidance or approach [23] might be considered in future research. Since crafting is an individual-level proactive behaviour, a better understanding of variables that can drive and motivate WNBC, such as self-efficacy [100], is of high interest for future research.

Fourth, our research did not uncover evidence supporting the transfer of WNBC-nonwork to enhanced work-related outcomes, such as work engagement [101]. This identifies a critical opportunity for future studies, which could aim to explore and extend mechanisms for effectively transferring resources from nonwork activities into improvements in work performance and job satisfaction by means of WNBC. For example, employee regeneration or recovery during nonwork time and, consequently, energy levels [102] may affect both degrees of crafting efforts and the effectiveness of life-domain transfers.

A further promising avenue, consistent with the principles of WNBC, involves examining the permeability of life-domain boundaries [91]. Adjusting these boundaries to be more fluid and permeable could facilitate a better exchange of resources and benefits between work and nonwork spheres, potentially leading to enhanced productivity, creativity, and overall work satisfaction. On the other hand, more detrimental effects like the association of WNBC-work and detachment from work, may increase when boundaries become more permeable. However, these different possibilities underscore the importance of understanding and managing the interplay between different life domains to optimize individual and organizational outcomes by crafting.

Fifth, future research should involve distinct research methods and study designs for replicating findings of the conducted study and structural equation model, e.g., diary studies on WNBC.

## Conclusion

The present study has contributed to the developing stream of crafting research beyond job crafting. The extent of crafting for work–nonwork balance depends on the demands and resource situations a person faces in both life domains. Work–nonwork balance crafting can contribute to a range of endpoints that can be seen as helpful and health-promoting to employees in fulfilling work tasks and caring for themselves and others in their work or nonwork lives.

### Electronic supplementary material

Below is the link to the electronic supplementary material.

Supplementary Material 1

Supplementary Material 2

## Data Availability

The datasets used and/or analysed during the current study available from the corresponding author [Philipp Kerksieck / philipp.kerksieck@uzh.ch] on reasonable request.

## Declarations

PK = Philipp Kerksieck, MK = Miika Kujanpää, JdB = Jessica de Bloom, RB = Rebecca Brauchli, GB = Georg F. Bauer.
