# Supplementary material for: A new perspective on balancing life domains: work–nonwork balance crafting
Source: BMC Public Health. 2024 Apr 22;24:1099. doi: 10.1186/s12889-024-18646-z (PMC11034155; doi:10.1186/s12889-024-18646-z)
Supplement: Supplementary file 1 — Supplementary Material 1 [file 12889_2024_18646_MOESM1_ESM.docx]

**Appendix A**

For the test of non-random sampling, we used multiple logistic regression (Goodman & Blum, 1996, p. 634) while the dependent variable was coded dichotomously, containing participants who either dropped out or participated in all three study waves. The models included all variables presented in the hypotheses as independent variables. Nagelkerke (NK) R^2^ indicated that the explained variance in all estimated models was not substantial; therefore, none of the predicting variables systematically contributed to non-random sampling: Job resources T1 (*B* = .10; *SE* = .07; *p* = .19; NK R^2^ = .00), job demands T1 (*B* = −.07; *SE* = .06; *p* = .25; NK R^2^ = .00), home resources T1 (*B* = −.01; *SE* = .06; *p* = .88; NK R^2^ = .00), home demands T1 (*B* = −.02; *SE* = .06; *p* = .80; NK R^2^ = .00), work nonwork balance crafting-work T2 (*B* = .03; *SE* = .10; *p* = .80; NK R^2^ = .00), work nonwork balance crafting-nonwork T2 (*B* = −.01; *SE* = .11; *p* = .92; NK R^2^ = .00).
